# Structural Comparison of *Enterococcus faecalis* and Human Thymidylate Synthase Complexes with the Substrate dUMP and Its Analogue FdUMP Provides Hints about Enzyme Conformational Variabilities

**DOI:** 10.3390/molecules24071257

**Published:** 2019-03-31

**Authors:** Cecilia Pozzi, Stefania Ferrari, Rosaria Luciani, Giusy Tassone, Maria Paola Costi, Stefano Mangani

**Affiliations:** 1 Department of Biotechnology, Chemistry and Pharmacy—Department of Excellence 2018-2020, University of Siena, via Aldo Moro 2, 53100 Siena, Italy; pozzi4@unisi.it (C.P.); giusy.tassone@unisi.it (G.T.); 2 Department of Life Sciences, University of Modena and Reggio Emilia, Via Campi 103, 41125 Modena, Italy; sferrari591@gmail.com (S.F.); rosaria.luciani@gmail.com (R.L.); mariapaola.costi@unimore.it (M.P.C.)

**Keywords:** thymidylate synthase, *Enterococcus faecalis*, half-site reactivity, x-ray structure, selectivity

## Abstract

Thymidylate synthase (TS) is an enzyme of paramount importance as it provides the only de novo source of deoxy-thymidine monophosphate (dTMP). dTMP, essential for DNA synthesis, is produced by the TS-catalyzed reductive methylation of 2′-deoxyuridine-5′-monophosphate (dUMP) using N^5^,N^10^-methylenetetrahydrofolate (mTHF) as a cofactor. TS is ubiquitous and a validated drug target. TS enzymes from different organisms differ in sequence and structure, but are all obligate homodimers. The structural and mechanistic differences between the human and bacterial enzymes are exploitable to obtain selective inhibitors of bacterial TSs that can enrich the currently available therapeutic tools against bacterial infections. *Enterococcus faecalis* is a pathogen fully dependent on TS for dTMP synthesis. In this study, we present four new crystal structures of *Enterococcus faecalis* and human TSs in complex with either the substrate dUMP or the inhibitor FdUMP. The results provide new clues about the half-site reactivity of *Enterococcus faecalis* TS and the mechanisms underlying the conformational changes occurring in the two enzymes. We also identify relevant differences in cofactor and inhibitor binding between *Enterococcus faecalis* and human TS that can guide the design of selective inhibitors against bacterial TSs.

## 1. Introduction

Thymidylate synthase (TS, EC 2.1.1.45) is a ubiquitous enzyme that catalyzes the reductive methylation of 2′-deoxyuridine-5′-monophosphate (dUMP) to 2′-deoxythymidyne-5′-monophosphate (dTMP) using N^5^,N^10^-methylenetetrahydrofolate (mTHF) as a cofactor. In cells, TS provides the only synthetic source of dTMP necessary for DNA biosynthesis. Indeed, TS inhibition halts the replication processes and induces apoptosis in rapidly dividing cells, which is an effect known as “thymineless death” [1]. Given its pivotal role in cell survival and proliferation, TS represents a validated target in anticancer and antibacterial chemotherapy; even though some bacteria express a further enzyme, the flavin dependent TS (FDTS), that is able to catalyze the same reaction. FDTS requires flavin adenine dinucleotide (FAD) and nicotinamide adenine dinucleotide phosphate (NADPH) to perform its action [2]. Important human pathogens, including *Bacillus anthracis*, *Clostridium botulinum*, and *Mycobacterium* species, encode for both TS and FDTS [2]. In other pathogenic strains, such as *Enterococcus faecalis*, only TS has been identified [2]. The development of TS inhibitors that can be used alone or in combination with FDTS targeting molecules is a promising strategy to generate new effective antibacterial drugs.

**Figure 1:**
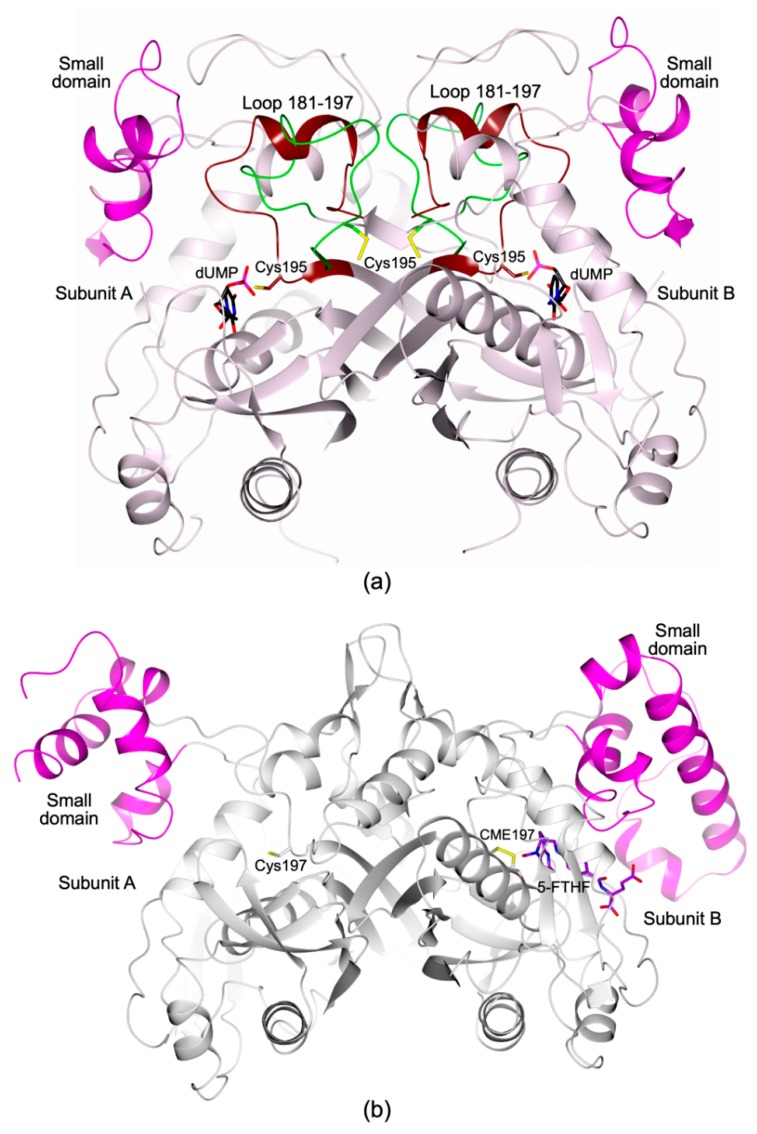
(**a**) Cartoon representation of the hTS homodimer in the active (PDB id 1HVY [3]) and inactive conformations (PDB id 1YPV [4]; for the inactive conformation, only the catalytic loop, including residues 181–197, is shown for clarity). In both subunits, the large and small domains are coloured light pink and dark pink, respectively. The two orientations of the catalytic loop (residues 181–197), defining the active (brown trace, PDB id 1HVY [3]) and inactive (green trace, PDB id 1YPV [4]) enzyme conformations, are displayed. The catalytic cysteine is shown in sticks (brown and green carbons in the active and inactive conformations, respectively). The position of the catalytic cavity is indicated by the presence of the substrate 2′-deoxyuridine 5′-monophosphate (dUMP, in sticks, black carbons). (**b**) Cartoon representation of the *Ef*TS homodimer (PDB id 3UWL [5]). In both subunits, the large and small domains are coloured white and dark pink, respectively. The large domain (residues 1–68 and 139–315), representing the conserved core of the enzyme, shows an α/β structure generated by seven α-helices and eight β-strands. The small domain (residues 69–138) are only fully structured (five α-helices connected by short loops) in the enzyme subunit with the cofactor binding site populated by 5-formyl-6-tetrahydrofolate (5-FTHF, in sticks, purple carbons). In the partner subunit, the small domain is highly flexible and only partially visible in the structures. The position of the active site is indicated by the catalytic Cys197 (in sticks, white carbons; in subunit B, it is modified as *S*,*S*-(2-hydroxyethyl)thiocysteine, CME197). In all figures, nitrogen atoms are coloured blue, oxygen red, sulphur yellow, and phosphorous magenta.

**Scheme 1:**
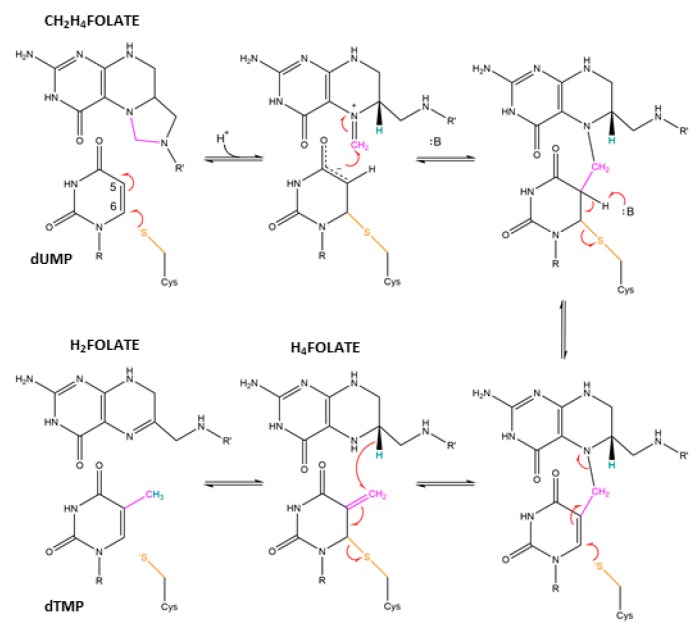
Proposed reaction scheme for the TS catalyzed reaction. Adapted from [9,10].

TS works as an obligate homodimer, having residues from both enzyme subunits that contribute to substrate binding in each catalytic site. TS subunits are characterized by two domains, known as large and small domains (LD and SD, respectively). The LD, showing a mixed α/β structure, represents the conserved core of the enzyme, whereas the SD is highly variable (in extension and structure) among the different organisms. The structures of TSs from various sources have been elucidated. Among them, only those from *Enterococcus faecalis* (*Ef*TS) and humans (hTS) have shown relevant domain changes that have been connected with different functional states of the enzymes (Figure 1) [3,4,5]. hTS switches between the active and inactive conformation through extended structural rearrangements that involve the catalytic loop (residues 181–197) [3,6]. In the active conformation, the catalytic Cys195 participates in the enzyme active site, whereas in the inactive conformation, it is exposed on the dimer interface (Figure 1a). On the other hand, *Ef*TS is characterized by the transition between the closed and open states (Figure 1b) [5]. In the former, the SD (residues 69–138) is fully ordered and contributes to create the cofactor binding site, whereas, in the latter, it is highly flexible and allows opening of the catalytic cavity (Figure 1b). Differently from the human enzyme, the open and closed states were identified within the same *Ef*TS homodimer, leading to asymmetry between the two enzyme subunits [5]. This phenomenon is related to the so-called “half-site reactivity”, whose presence in all forms of TS is still matter of debate [7,8]. The transitions to the closed state in *Ef*TS, and to the active conformation in hTS, are fundamental to create functional active sites in which dUMP can bind, followed by the cofactor [3,5]. According to the reaction mechanism, the thiol of the conserved catalytic cysteine (Cys197 and Cys195, in *Ef*TS and hTS, respectively) attacks the carbon atom in position 6 (C6) on the nucleotide pyrimidine base. This leads to the formation of a covalent adduct (Scheme 1) [9,10]. The dUMP uracil carbon in position 5 (C5) is then activated to accept the methyl moiety (C11) and the hydride donated by mTHF.

In the present work, we report the structural characterization of *Ef*TS in complex with the substrate dUMP and its analogue 5-fluoro-2′-deoxyuridine-5′-monophosphate (FdUMP). FdUMP is a known potent TS inhibitor [11], for which we measured a K_i_ towards *Ef*TS in the nM range. In both structures, dUMP and FdUMP populate only one catalytic cavity of the enzyme dimer, providing new evidence to support the half-site reactivity model proposed for this TS enzyme [5]. Furthermore, the comparison with the structure of native *Ef*TS shows that substantial changes are induced by substrate binding. The structural analysis was also extended to the human enzyme (48 % sequence identity), reporting its characterization in complex with the same molecules. Even though former studies described the interactions of FdUMP within the hTS active site [12,13], we observe significant differences in the inhibitor binding mode. The structural information achieved provides new clues to explain the mechanisms guiding the conformational switches in these enzymes, highlighting a prominent role of the dimer interface in inter-subunit communication. Furthermore, the structural comparison of human and *Ef*TS shows the presence of non-conserved residues in their catalytic cavities, mainly involved in cofactor binding. This information is of key importance for the rational development of inhibitors able to selectively target *Ef*TS without altering the activity of the counterpart enzyme in the human host.

## 2. Results and Discussion

### 2.1. EfTS in Complex with the Substrate dUMP and Its Analogue FdUMP

#### 2.1.1. *Ef*TS Overall Fold

**Figure 2:**
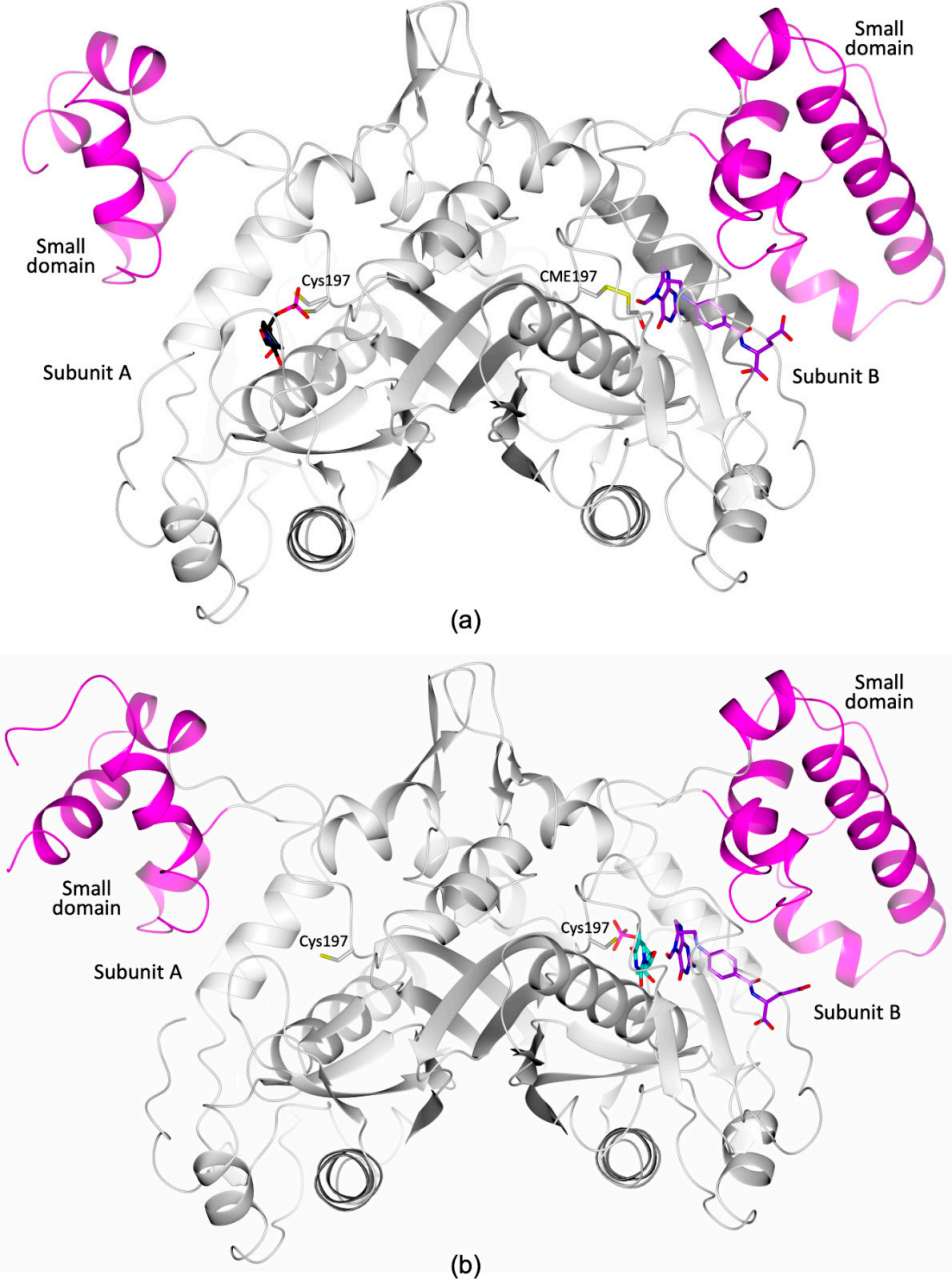
Dimer of *Ef*TS (large and small domains are represented as grey and dark pink cartoon, respectively) in complex with (**a**) dUMP (in sticks, black carbons) and (**b**) FdUMP (in sticks, cyan carbons). Structural heterogeneity within the functional *Ef*TS dimer is observed in both complexes. In the *Ef*TS–dUMP complex, the nucleotide is bound to subunit A, whereas the cofactor-like molecule 5-formyl-6-tetrahydrofolate (5-FTHF, in sticks, purple carbons) occupies the active site of subunit B in which the catalytic cysteine is modified as *S*,*S*-(2-hydroxyethyl)thiocysteine (CME197, in sticks, grey carbons). On the other hand, in the *Ef*TS–FdUMP complex, the substrate-like inhibitor (in sticks, cyan carbons) is bound to subunit B together with the cofactor-like molecule 5-FTHF (in sticks, purple carbons).

*Ef*TS has the constitutive dimeric quaternary structure typical of all TS enzymes [14]. The two independent *Ef*TS dimers (A-B and C-D), present in the cell asymmetric unit (ASU), have essentially identical structures and show structural heterogeneity between subunits due to asymmetric ligand binding within the enzyme active sites (Figure 2). The structure of the “as prepared” *Ef*TS (PDB id 3UWL), formerly characterized by us, revealed the presence of a folate derivative, constitutively populating one active site within each *Ef*TS homodimer [5]. This cofactor analogue, interpreted as 5-formyl-6-tetrahydrofolate (5-FTHF), copurifies with the enzyme and generates asymmetry between the two enzyme protomers (*vide infra*, Figure 2). 5-FTHF is a metabolite of the folate cycle that naturally occurs in the cell [15]. In the homodimer, Arg177 and Arg178 from one *Ef*TS subunit participate in the assembly of the active site of the partner subunit. In both complexes, subunits B and D are in the closed state, displaying the SD (residues 69–138) fully structured in five α-helices connected by short loops, with the active site hosting the 5-FTHF molecule (Figure 2). On the other hand, the partner subunits (A and C) adopt the open conformation, showing the SD partially ordered in only three α-helices. Due to disorder, residues 92–124 and 98–118, in the dUMP and FdUMP complexes, respectively, have been excluded from our models.

#### 2.1.2. *Ef*TS–dUMP Complex

**Figure 3:**
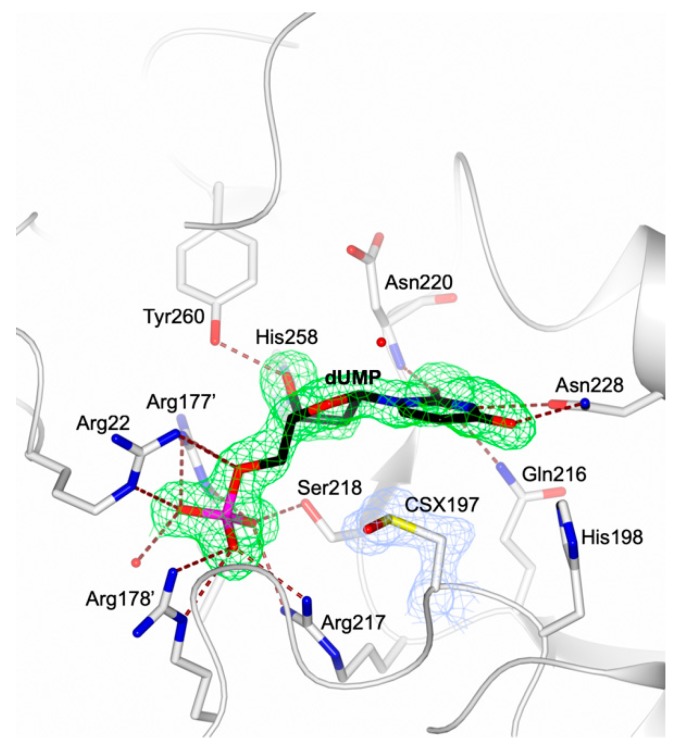
Active site view of *Ef*TS (white cartoon and carbon atoms) in complex with dUMP (in sticks, black carbon atoms). In the panel, dUMP is surrounded by the omit map (green mesh) contoured at the 2.5 σ level. Key interactions (salt bridges, hydrogen and halogen bonds) are displayed as tan dashed lines. The catalytic cysteine is oxidized to sulfenic acid (S-oxycysteine, CSX197), surrounded by the *2Fo-Fc* map (blue mesh, contoured at the 1.5 σ level).

The structure of *Ef*TS in complex with the substrate dUMP was determined to be 1.75 Å resolution (Figure 2, Table S1). The cofactor-analogue (6*S*)-5-formyl-6-tetrahydrofolate (5-FTHF) is entrapped in the active sites of subunits B and D by the same tight network of interactions already observed in the substrate-free form of the enzyme (Figure S1) [5]. Moreover, in these subunits, the catalytic Cys197 is modified as *S*,*S*-(2-hydroxylethyl)thiocysteine (CME197, Figure 2a and Figure S1) by the reaction with β-mercaptoethanol (added to the protein buffer during the purification process [5]). In the partner subunits (A and C), the electron density of the catalytic Cys197 suggests that it had been oxidized to sulfenic acid (*S*-oxycysteine, CSX197 in Figure 3). Despite the oxidation of Cys197, the substrate dUMP populates the active site, showing its uracil moiety positioned just above the cysteine sulphur (C6–Sγ distance of ~3.7 Å) (Figure 3). In both chains, the substrate shows the same network of interactions (Figure 3). The dUMP phosphate is salt-bridged to Arg22, Arg217, Arg177’, and Arg178’ (the last two from the partner subunit) and it is further H-bonded to the hydroxyl of Ser218. The dUMP ribose hydroxyl is H-bonded to His258 and Tyr260. The uracil ketone oxygen in position 2 receives H-bonds from the backbone nitrogen of Asn220 and the amide nitrogen of Gln216. Furthermore, the amide moiety of Asn228 donates an H-bond to the uracil ketone in position 4 and receives an H-bond from the uracil nitrogen in position 3. 

**Figure 4:**
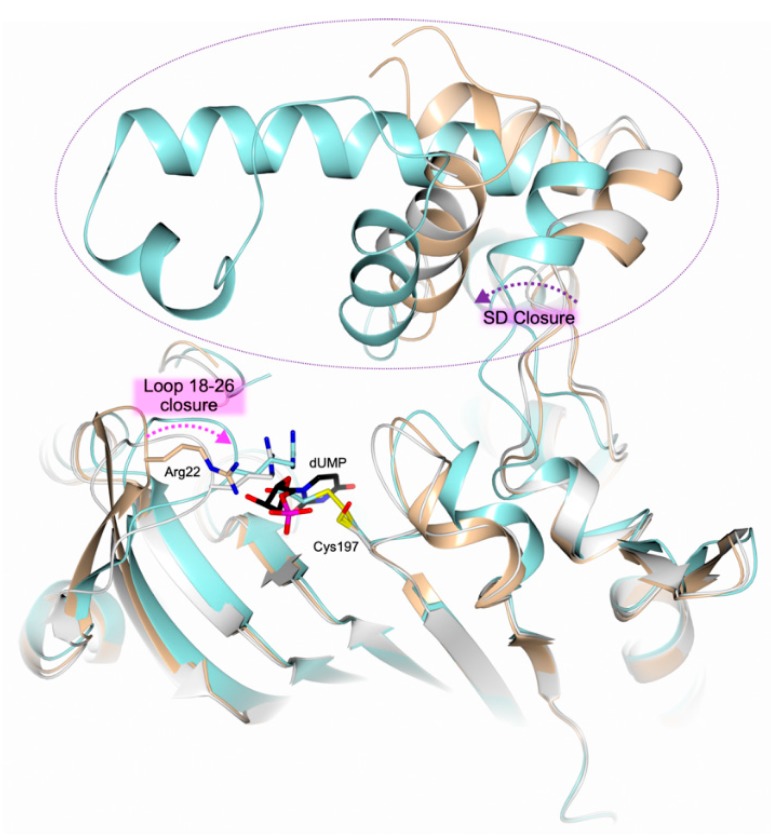
Structural comparison of the different states observed in the structure of substrate-free *Ef*TS (PDB id 3UWL [5], open conformation in gold and closed conformation in light blue) and in its complex with dUMP (open conformation in white, only the dUMP-bound subunit is shown). The closed conformation of both SD (circled) and loop 18–26 is observed in subunits B and D of substrate-free *Ef*TS (light blue cartoon and carbon atoms) where 5-FTHF is bound. The open conformation of SD and loop 18-26 is observed in chains A and C of the substrate-free enzyme (gold cartoon and carbons). In the *Ef*TS–dUMP complex (in white), a closed conformation of loop 18–26 is visible in the dUMP-bound subunits. In *Ef*TS, the open/closed transition occurs as a biphasic process in which the dUMP binding triggers the closure of loop 18–26 (pink dashed arrow), whereas the binding of the cofactor (or analogue) induces the movement and the organization of the whole SD (purple dashed arrow).

While subunits A and C where dUMP is bound display the open conformation, subunits B and D where 5-FTHF is bound are in the closed conformation. The structural comparison with the substrate-free subunits of the enzyme (PDB id 3UWL [5]) shows that meaningful changes occur upon dUMP binding (Figure 4). The most evident rearrangement concerns the flexible loop 18–26, which moves towards the catalytic cavity in the dUMP-bound subunits, exposing Arg22 inside the phosphate recognition pocket (Figure 4). The movement of Arg22, whose Cα shifts by ~5.8 Å, is fundamental to create the four-arginine cluster that anchors the dUMP phosphate in the substrate site. However, this conformation of the 18–26 loop resembles that observed in the closed state of the substrate-free enzyme (PDB id 3UWL), where 5-FTHF is bound and in which the four-arginine cluster is fully formed and binds a sulfate/phosphate anion [5] (Figure 4). On the contrary, in the subunits of the substrate-free enzyme in the open conformation (subunits A and C of 3UWL), the loop is turned away from the active site and Arg22 points to the solvent-exposed surface of *Ef*TS (Figure 4). 

Remarkably, despite the full occupancy of the substrate site by dUMP and the movement of the 18–26 loop towards dUMP, the SD retains the open conformation, in which it is widely unstructured. This observation strongly suggests that in *Ef*TS, the open/closed transition occurs as a biphasic process in which the dUMP accommodation triggers the closure of the loop 18–26, whereas the binding of the cofactor (or analogue) induces the movement and the organization of the whole SD.

#### 2.1.3. *Ef*TS–FdUMP Complex

**Figure 5:**
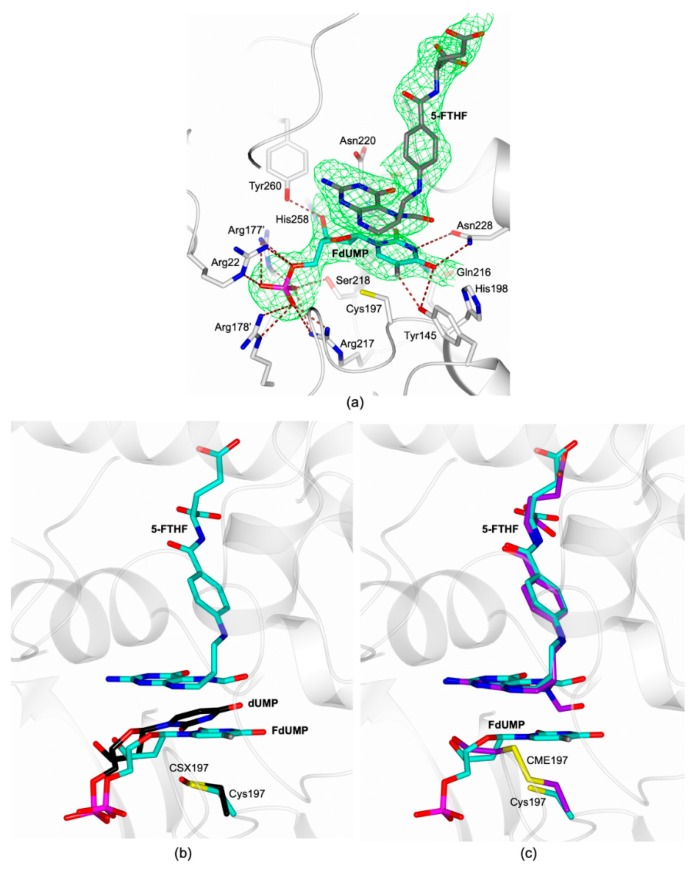
(**a**) Active site view of *Ef*TS (white cartoon and carbon atoms) in complex with FdUMP (in sticks, cyan carbons) and 5-FTHF (in sticks, dark grey carbons). In the panel, FdUMP and 5-FTHF are surrounded by their omit map (green mesh) contoured at the 2.5 σ level. Key interactions (salt bridges, hydrogen and halogen bonds) are displayed as tan dashed lines. (**b**) View of the active site of *Ef*TS in complex with dUMP (dUMP and CSX197 in sticks, black carbons) superimposed to the *Ef*TS-FdUMP-5-FTHF complex (FdUMP, Cys197, and 5-FTHF in sticks, cyan carbons). (**c**) View of the active site of the *Ef*TS–FdUMP-5-FTHF complex (FdUMP, Cys197, and 5-FTHF in sticks, cyan carbons) superimposed to the substrate-free *Ef*TS (PDB id 3UWL, [5]; Cys197 modified as *S*,*S*-(2-hydroxyethyl)thiocysteine, CME-197 and 5-FTHF in sticks, purple carbons). Fluorine atoms are colored grey.

Inhibition assays of *Ef*TS activity performed on FdUMP resulted in a K_i_ of 74 nM. The structure of *Ef*TS in complex with the substrate analogue FdUMP was determined at 2.88 Å resolution (Table S1), consistently showing subunit asymmetry within each enzyme dimer (Figure 2b). FdUMP populates the active sites of subunits B and D (Figure 5a) turned to a closed state, whereas chains A and C remain in the open conformation typical of ligand-free subunits (Figure 2b) [5]. In subunits B and D, FdUMP binding is accompanied by the accommodation of 5-FTHF in the cofactor site (the same cofactor analogue also entrapped in the active site of the native enzyme [5]; Figure 5a). FdUMP occupies the substrate pocket, where it mimics the dUMP binding mode, forming similar interactions within the cavity (Figure 3 and Figure 5a). Nonetheless, the 5-fluoro-uracil moiety of FdUMP is positioned closer to the catalytic Cys197 (distance of ~2.7 Å between the inhibitor C6 and the cysteine thiol) (Figure 5b). The shortening of this distance is possibly related to the different oxidation states of the catalytic cysteine, observed in the two complexes. Notably, in the dUMP-complex, the Cys197 side chain is modified to sulfenic acid, thus blocking the nucleophilic attack of the thiol to the substrate C6. On the contrary, in the complex with FdUMP, the thiol of Cys197 is reactive and can form a covalent adduct with the inhibitor that, indeed, is positioned closer to the catalytic residue. In this arrangement, the 5-fluorine on the FdUMP uracil moiety is proximal (~2.7 Å) to the Tyr145 hydroxyl, suggesting the formation of a halogen bond with it [16] (Figure 5a). Moreover, the same moiety of Tyr145 donates an H-bond to the FdUMP ketone oxygen in position 4, contributing to stabilize the inhibitor within the catalytic cavity.

At variance with the dUMP-complex, FdUMP binding is further stabilized by 5-FTHF (Figure 5a). Indeed, the pteridin moiety of 5-FTHF is accommodated just above the 5-fluoro-uracil of the substrate analogue, placing its 5-formyl substituent proximal to the FdUMP C5 (distance of ~3.2 Å). This configuration of the active site mimics the stage of the catalytic reaction in which the methyl moiety on the cofactor N5 (resulting from the aperture of the mTHF five-membered ring) is ready to be transferred to the substrate [9]. The structural comparison of the present ternary complex with the “as prepared” *Ef*TS, where 5-FTHF alone populates the active site of one dimer subunit [5], shows that the binding mode of 5-FTHF is altered by bound FdUMP (Figure 5c). Notably, in the former *Ef*TS structure, the catalytic Cys197 is modified to *S*,*S*-(2-hydroxylethyl)thiocysteine by the reaction with β-mercaptoethanol, allowing the 5-FTHF formyl moiety to protrude in the uracil pocket (Figure 5c). In contrast, the binding of FdUMP to the substrate site induces a rearrangement of 5-FTHF, whose 5-formyl group shifts by ~1.5 Å away from the inhibitor FdUMP (Figure 5c).

### 2.2. hTS in Complex with the Substrate dUMP and Its Analogue FdUMP

#### 2.2.1. hTS Overall Fold

hTS was crystallized in the low-salt conditions (see Materials & Methods section; [4]), in the presence of either dUMP or FdUMP. In both complexes, the two subunits of the hTS homodimer adopt the active conformation, showing that Cys195 is exposed to the catalytic cavity, as occurs in previously reported structures [3,17]. The one and half homodimers found in the ASU were fully traced, apart for the first twenty-five N-terminal residues (and the twelve residues belonging to the non-removable His^6^-tag). 

#### 2.2.2. hTS–dUMP Complex 

**Figure 6:**
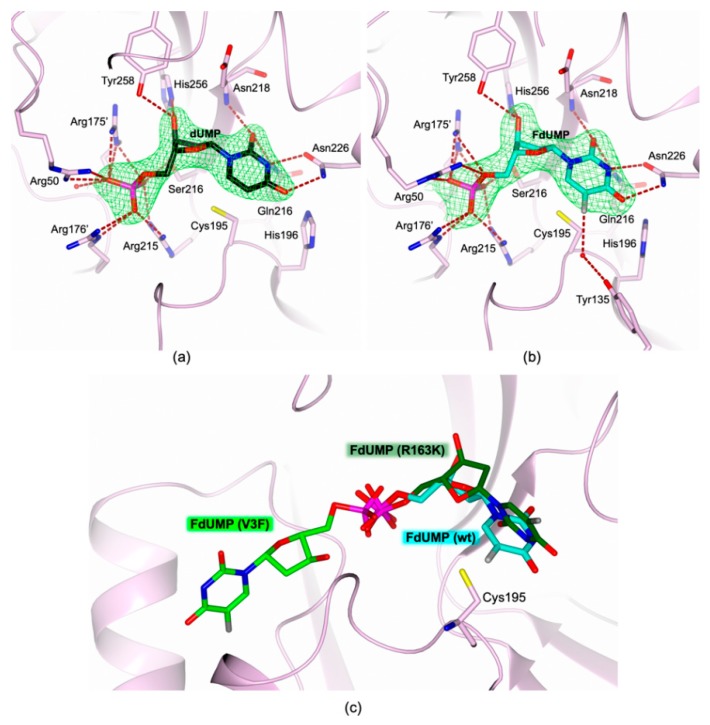
(**a**,**b**) Active site view of hTS (light pink cartoon and carbon atoms) in complex with (**a**) dUMP (in sticks, black carbon atoms) and (**b**) FdUMP (in sticks, cyan carbons). In both panels, the ligands are surrounded by the omit map (green mesh) contoured at the 2.5 σ level, and key interactions (salt bridges, hydrogen and halogen bonds) are displayed as tan dashed lines. (**c**) Different orientations of FdUMP in the complexes with the wild-type hTS (pink cartoon and carbon atoms, FdUMP in sticks, cyan carbons) and the variants R163K (FdUMP in sticks, dark green carbons; 3H9K [13]) and V3F (FdUMP in sticks, green carbons; PDB id 3EJL [12]).

The structure of hTS in complex with dUMP was determined to 1.95 Å resolution (Table S1), providing a clear picture of substrate binding within the catalytic cavity (Figure 6a). The dUMP phosphate is salt bridged to the four arginines, Arg50, Arg215, Arg175’, and Arg176’ (the last two from the partner subunit), and it further receives an H-bond from Ser216 (Figure 6a). The dUMP ribose hydroxyl is H-bonded to His256 to Tyr258. The uracil ketone oxygen in position 2 receives two H-bonds from the backbone nitrogen of Asn218 and the amide nitrogen of Gln216. Nearby, the amide of Asn226 receives an H-bond from the dUMP uracil N3 and donates one to the ketone O4. The thiol of the catalytic Cys195 is located in an optimal position to attack the uracil C6 of the substrate (interatomic distances ranging from 3.14 Å to 3.27 Å in the three enzyme subunits). The dUMP binding mode and its interactions within the catalytic cavity are conserved with respect to those formerly described in binary and ternary complexes of hTS [17].

#### 2.2.3. hTS–FdUMP Complex 

The structure of hTS in complex with the substrate analogue FdUMP (K_i_ of 1.1 nM [18]) was determined to 2.08 Å resolution (Table S1). FdUMP fully occupies the hTS active site, perfectly mimicking the interactions entailed by dUMP in the substrate pocket (Figure 6). The 5-fluorine of FdUMP forms an additional water-mediated interaction with the Tyr135 hydroxyl (Figure 6b). FdUMP C6 is positioned 3.09–3.29 Å away from the Cys195 thiol, which is the same distance observed in the dUMP complex. Former structural analysis of the hTS variant R163K, showed that FdUMP adopts a slightly different orientation inside the substrate pocket (PDB id 3H9K [13]) (Figure 6c). Both the ribose and the 5-fluoro-uracil moieties of FdUMP are rotated by ~40° with respect to the orientations observed by us in the complex with the wt-enzyme, resulting in a remarkable loss of interactions with the surrounding residues inside the cavity (Figure 6c). Due to this rotation, the FdUMP C6 moves away from the Cys195 thiol in the R163K variant (distance of ~4.7 Å), far from the productive position, leading to the formation of the covalent adduct. Reasonably, the mutation of residue 163, located ~16 Å away from the catalytic cavity, is not responsible for these changes. Additionally, the hTS V3F variant-FdUMP complex has been reported (PDB id 3EJL [12]), showing that the substrate analogue is rotated outside the catalytic cavity (Figure 6c). In this configuration, FdUMP is directed towards the solvent-exposed surface of the enzyme with C6 placed ~12.0 Å away from the catalytic cysteine. The FdUMP binding modes observed in the complexes of the above hTS variants are not fully consistent with the mechanism of action of the inhibitor, relying on the formation of a covalent adduct with the TS catalytic cysteine [19]. On the other hand, in our complex, FdUMP mimics the substrate, placing its 5-fluoro-uracil C6 in an optimal position to form the covalent adduct with the reactive thiol of Cys195.

### 2.3. Structural Comparison between Bacterial and the Human TSs in Complex with dUMP and FdUMP

**Figure 7:**
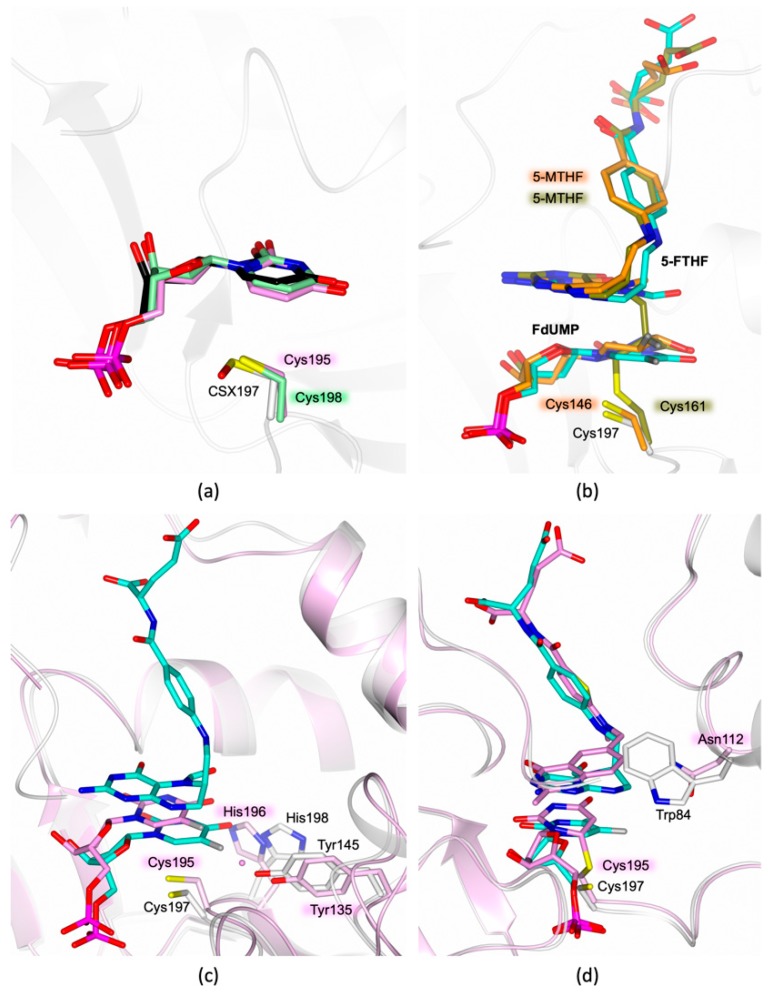
Active site view of the superimposition among the complexes: (**a**) *Ef*TS–dUMP (CSX197 in sticks, white carbon atoms; dUMP in sticks, black carbons), *Lc*TS–dUMP (in sticks, light green carbons; PDB id 2TDM [21]), and hTS–dUMP (in sticks, pink carbons). (**b**) *Ef*TS–FdUMP–5-FTHF (Cys197 in sticks, white carbon atoms; ligands in sticks, cyan carbons), *Mtb*TS–FdUMP–5-methyl-THF (in sticks, orange carbons; PDB id 4FOG, unpublished results), and *Bs*TS–FdUMP–5-methyl-THF (in sticks, olive carbons; PDB id 1B02 [22]). (**c**) *Ef*TS–FdUMP–5-FTHF (white cartoon, Cys197 in sticks, white carbon atoms; ligands in sticks, cyan carbons) and hTS–FdUMP (pink cartoon and carbon atoms). (**d**) *Ef*TS–FdUMP–5-FTHF (white cartoons, Cys197 white carbon atoms; ligands in sticks, cyan carbons) and hTS–dUMP–raltitrexed (pink cartoon and carbon atoms). In the (**a**) and (**b**) panels, only the catalytic cysteine is shown in the structures of *Lc*TS, hTS, *Mtb*TS, and *Bs*TS for clarity.

The structures of several bacterial TS–substrate complexes have been reported in the PDB (*Escherichia coli* TS, *Ec*TS, PDB id 1BID [20]; *Lactobacillus casei*, *Lc*TS, PDB id 2TDM [21]; *Staphylococcus aureus* TS, *Sa*TS, PDB id 4DQ1 (unpublished results); *Mycobacterium tuberculosis* TS, *Mtb*TS, PDB id 3QJ7 (unpublished results); *Corynebacterium glutamicum* TS, *Cg*TS, 4H0U (unpublished results). The sequence alignment is displayed in Figure S2) and shows a remarkable conservation of the residues involved in dUMP binding. Figure 7a shows the superimposition of dUMP in *Ef*TS, *Lc*TS, and hTS, highlighting the same pose of the substrate in all enzymes. At variance with the other complexes, in *Ef*TS, the catalytic Cys197 is oxidized in those subunits where dUMP is bound, evidencing that the residue modification does not alter the ability of the enzyme to accommodate the substrate. However, this explains the tiny gain in the distance between the uracil C6 and the cysteine thiol, resulting in ~3.7 Å in *Ef*TS, whereas distances of 3.2–3.4 Å characterize other bacterial TS–dUMP complexes [20,21]. In all complexes, the four-arginine cluster, anchoring the dUMP phosphate, is highly conserved. This evidence strongly suggests that the closure of the loop 18–26, observed in *Ef*TS upon dUMP binding, is important to stabilize the substrate inside the active site. 

On the other hand, a slightly different configuration is observed in the complex with the substrate analogue FdUMP, which populates the catalytic cavity of *Ef*TS together with the cofactor derivative 5-FHTF (Figure 7b). The ternary complex observed in *Ef*TS is remarkably similar to the structure of *Mtb*TS in complex with FdUMP and the cofactor 5-methyl-THF (deriving from aperture of the mTHF 5-membered ring; PDB id 4FOG, unpublished results) (Figure 7b). FdUMP adopts the same binding mode in both complexes, showing its uracil C6 positioned ~2.7 Å away from the thiol of the catalytic cysteine (Cys197 in *Ef*TS, Cys146 in *Mtb*TS). However, the 5-fluorine of the inhibitor is observed in two different orientations, being directed towards the cofactor in *Mtb*TS, whereas in *Ef*TS, it points towards the hydroxyl of Tyr145, allowing the formation of a halogen bond with it. Conserved binding modes also characterize the cofactor 5-methyl-THF and its analogue 5-FTHF, bound to *Mtb*TS and *Ef*TS. In each instance, the pterin N5 is positioned on top of FdUMP uracil C5. On the other hand, a slightly different configuration inside the catalytic cavity is displayed by the analogous complexes (FdUMP and 5-methyl-THF) obtained with *Bacillus subtilis* TS (*Bs*TS; PDB id 1B02 [22]) and *E. coli* TS (PDB id 1TLS [23]). The structure of *Bs*TS is reported in Figure 7b. In both structures, the formation of the ternary covalent adduct is observed, resulting from the covalent anchoring of the FdUMP C6 to the cysteine thiol and of the FdUMP C5 to the cofactor 5-methyl moiety. In the ternary covalent adducts, both ligands are slightly distorted. In *Ef*TS, 5-FTHF, having a 5-formyl group that replaces the methyl of the cofactor, acts as an inhibitor, preventing the formation of the ternary covalent adduct. Therefore, our *Ef*TS-FdUMP-5-FTHF ternary complex mimics the pre-reactive step of catalysis in which both substrate and cofactor are aligned in the cavity to allow the transfer of the pterin N-5 substituent.

The structure of the *Ef*TS–FdUMP complex was also compared to that of the human enzyme, evidencing the occurrence of tiny changes in the inhibitor binding mode (Figure 7c). In *Ef*TS, the FdUMP 5-fluorine is close to the Tyr145 hydroxyl, forming a halogen bond with it (Figure 5a and Figure 7c). In contrast, in the hTS complex, the FdUMP uracil is shifted far from the isostructural Tyr135, and a water molecule mediates its interaction with the fluorine of the inhibitor (Figure 6b and Figure 7c). A comparison of *Ef*TS and hTS complexes shows that, in hTS, the orientation of His196 prevents the inhibitor from lying closer to Tyr135, whereas in *Ef*TS, the sidechain of His198 (corresponding to hTS His196) is rotated by ~70°, allowing the direct interaction between the fluorine and Tyr145 (Figure 7c). The superimposition further evidences that FdUMP in hTS partially overlaps the cofactor site of *Ef*TS. In addition, hTS Cys195 is slightly moved with respect to the position of the corresponding residue observed in *Ef*TS. To explain these differences, the structure of *Ef*TS was compared to the that of hTS in complex with dUMP and the cofactor analogue raltitrexed (PDB id 1HVY [3]), evidencing that even the cofactor site is somewhat shifted in the human enzyme (Figure 7d). Notably, in *Ef*TS, the indole system of Trp84, belonging to the SD, protrudes inside the catalytic cavity and establishes van der Waals interactions with the cofactor analogue, generating a hindrance that induces a more compact assembly of FdUMP and 5-FTHF within the active site (Figure 7d). In hTS, Asn112 matches the position *Ef*TS Trp84, allowing a less tight configuration inside the catalytic cavity (Figure 7d). This tryptophan residue of *Ef*TS is conserved among bacterial TSs of other human pathogens, including *S. aureus*, *B. melitensis*, and *Mycobacterium tuberculosis* (Figure S2). The presence of Trp in bacterial TS versus Asn in the active site of hTS can be of key importance for the design of selective bacterial TS inhibitors. 

### 2.4. Conformational Transitions in EfTS and hTS Enzymes

The structural studies performed on both *E. faecalis* and human TS highlighted the existence of different stable conformations that were connected to distinct functional states of the enzymes. Even though various hypotheses have been formulated, the mechanisms behind these conformational transitions have not been fully elucidated. In *Ef*TS, the open and closed states characterize the two subunits of a single protein dimer (asymmetric homodimers), suggesting that this enzyme works through half-site reaction mechanisms [5]. Half-site reactivity, although with much less evident conformational changes, has also been reported for other bacterial TSs that differ in structure from *Ef*TS [7,8]. On the other hand, conformational transitions are also characteristic of the human enzyme in which the active and inactive conformations are instead concomitantly adopted by both enzyme subunits, resulting in a symmetric homodimer. 

The characterization of *Ef*TS in complex with dUMP provides evidence that the transition between the open and the closed state occurs in this enzyme as a bi-phasic process. dUMP binding inside the catalytic cavity triggers the closure of the loop 18–26, exposing Arg22 inside the catalytic site, in which it participates in the four-arginines cluster that anchors the dUMP phosphate group. Remarkably, the binding of the substrate does not influence the whole SD that keeps the open conformation. Moreover, the movement of the 18–26 loop does not affect the partner subunit, where 5-FTHF is bound, which adopts the closed conformation. The flexibility of the 18–26 loop is a peculiar feature of *Ef*TS; indeed, there is no evidence of analogous rearrangements in other TS enzymes.

On the other hand, the conformational transitions that characterize hTS do not involve loop 26–54, corresponding to the *Ef*TS loop 18–26, which is oriented towards the catalytic cavity in both hTS active and inactive conformations. hTS is characterized by the high flexibility of the catalytic loop (residues 181–197) that shifts between the active and the inactive conformations. In the inactive conformation, the catalytic Cys195 moves out from the catalytic cavity into the enzyme dimer interface. The active conformation of the hTS catalytic loop closely resembles the orientation of the corresponding residues 183–199 in *Ef*TS. It is worth noting that conformational transitions in both enzymes involve the SD, which shifts from a flexible to ordered structure. Both the open state of *Ef*TS and the inactive conformation of hTS are characterized by widely unstructured SDs, which become fully orderered after the transition to the closed state and active conformation, respectively. 

**Figure 8:**
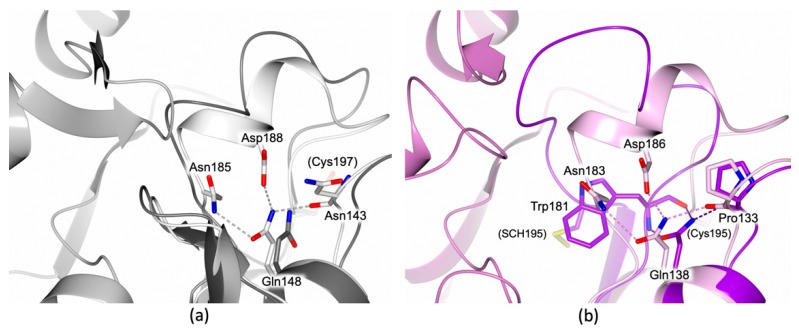
(**a**) Structural superimposition between the closed (white cartoon and carbon atoms; H-bonds as light grey dashed lines) and the open (dark grey cartoon and carbons) states of *Ef*TS. (**b**) Structural comparison of the active (pink cartoon and carbon atoms, H-bonds as pink dashed lines) and the inactive (purple cartoon and carbon atoms, H-bonds as purple dashed lines) conformations of hTS.

The structural comparison of these enzymes further evidences a common orientation for Gln148 in the *Ef*TS closed state and for the corresponding Gln138 in the hTS active conformation (Figure 8). Notably, the side chains of both residues flip by ~180° following the transition to the open state and inactive configurations (Figure 8). In the *Ef*TS closed state, the amide moiety of Gln148 is H-bonded to Asn143, Asn185, and Asp188 (Figure 8a). All these interactions are lost in the open state, in which the side chain of Gln148 is rotated away from these residues (Figure 8a). In *Ef*TS, Asn185 and Asp188 belong to a loop located at the dimer interface, whose placement is also affected by the enzyme conformational transitions. Indeed, in the open state, Gln148 rotation releases the loop 183–199, which shifts towards the partner subunit by ~2.7 Å, whereas it is tied to Gln148 in the closed state. Furthermore, the *Ef*TS residues 183–199 directly participate in the interface interaction with the partner subunit, suggesting that they can be involved in the inter-subunit communication, regulating the half-site reactivity that characterize this enzyme. 

Analogously to the closed state of *Ef*TS, in the active conformation of hTS, Gln138 forms conserved interactions with Pro133, Asn183, and Asp186 (Figure 8b). In contrast, the transition to the inactive conformation induces a flip of the Gln138 amide moiety, which loses its interactions with residues 183 and 186, but forms H-bonds with the backbone of Trp181 (Figure 8b). In hTS, residues 181–197 belong to the catalytic loop, highlighting the importance of the rearrangement of the Gln138 side chain to stabilize both conformations. It is worth noting that the portion 181–190 participates in the dimer interface, suggesting that the mobility of the catalytic loop also mediates the inter-subunit communication in hTS.

## 3. Materials and Methods 

### 3.1. Macromolecule Production 

*Ef*TS was prepared according to an established procedure [5]. Human TS was expressed and purified as previously described, with minor modifications [24]. Briefly, hTS was expressed as the His^6^-tag protein (the not-cleavable N-terminal His^6^-tag was encoded by the pQE-80L expression plasmid) in *E. coli* strain BL21(DE3). Bacteria were cultured at 30 °C in the auto-induction medium ZYP-5052 [25] for 30 h. Cells, harvested by centrifugation (3000× *g*, 15 min, 8 °C), were suspended in buffer A (50 mM HEPES, pH 7.5, and 30 mM NaCl), with the addition of 20 mM imidazole, 0.2 mM phenylmethylsulfonyl fluoride (PMSF), and 0.5 mg mL^−1^ lysozyme, and then disrupted by sonication after 60 min incubation on ice. The cell-free extract, obtained by centrifugation (12,000× *g*, 60 min, 8 °C), was applied to a HisTrap HP 5 mL column (GE Healthcare, Chicago, IL, USA) and eluted using a 250–500 mM imidazole concentration in the same buffer. Fractions containing the target protein were pooled and dialyzed in buffer A. The high purity (>98%) of the resulting protein sample was confirmed by SDS-PAGE analysis and MALDI-TOF mass spectrometry.

### 3.2. Enzyme Activity and Inhbition Assayes 

Enzyme activity assays were performed spectrophotometrically, according to a reported protocol [5,24]. Briefly, 1 mL reaction mixtures were prepared by adding aliquots of the enzyme (80–120 nM) to the assay buffer (50 mM TES, pH 7.4, 25 mM MgCl_2_, 6.5 mM HCHO, 1 mM EDTA, 75 mM β-mercaptoethanol), including variable concentrations of dUMP (50–200 μM) and mTHF (50–200 μM). Reactions, started by the addition of the substrate, were monitored by following the increase in absorbance at 340 nm during the oxidation reaction of mTHF to 7,8-dihydrofolate (DHF), for 3 min. Kinetic parameters characterized for both enzymes were in complete agreement with those formerly reported [5,24]. Inhibition assays for FdUMP were performed under the above condition using an enzyme concentration of 95 μM, and substrate and cofactor concentrations of 120 and 50 μM, respectively. The inhibition data versus hTS were already reported in the literature and were confirmed by our experiments [18]. The dose-response curve for *Ef*TS inhibition by FdUMP was generated by adding compound concentrations ranging from 0.25 to 10 μM. The IC_50_ value, obtained by non-linear regression fitting of the initial rates (in terms of residual enzyme activity) versus the logarithm of the inhibitor concentrations, resulted in 0.815 ± 0.099 μM (as calculated using the software GraphPad Prism 8, San Diego, CA, USA). Measures were performed in triplicate. The K_i_ value of 74 nM for FdUMP versus *Ef*TS was calculated using the equation IC_50_ = K_i_ (1 + S/K_M_) [26] (where S and K_M_ are the substrate concentration and the Michaelis-Menten constant, respectively).

### 3.3. Protein Crystallization 

#### 3.3.1. *Ef*TS 

*Ef*TS crystallization was performed using the hanging drop vapour-diffusion method [27] at room temperature. Crystals of the complex *Ef*TS–dUMP were grown in drops prepared by mixing 4 μL of sample (*Ef*TS 7 mg mL^−1^ in 25 mM phosphate buffer, pH 6.8, 100 mM dUMP, and 2 mM DTT) and 2 μL of precipitant (2.2–2.5 M ammonium sulfate and 0.1 M HEPES, pH 7.5) solutions and equilibrated over an 800 μL reservoir. Crystals of *Ef*TS in complex with FdUMP were grown using the Nextal Crystallization Tool [28]. Drops were prepared by mixing equal volumes of sample (*Ef*TS 7 mg mL^−1^ in 25 mM phosphate buffer, pH 6.8, 4 mM FdUMP, and 2 mM DTT) and the above precipitant solution and were equilibrated over a 1 mL reservoir. Before data collection, crystals (grown within two-three weeks) were transferred to the cryoprotectant solution (20 % *vol*/*vol* ethylene glycol, 2.5 M ammonium sulfate and 0.1 M HEPES, pH 7.5) and flash frozen in liquid nitrogen.

#### 3.3.2. hTS 

Crystals of hTS were grown at room temperature using the hanging-drop vapor-diffusion method [27]. Drops were prepared by mixing equal volumes of sample (hTS 20 mg mL^−1^ in 50 mM HEPES, pH 7.5, 30 mM NaCl, 20 mM β-mercaptoethanol, and either 40 mM dUMP or 2 mM FdUMP) and precipitant (27–30 % *wt*/*vol* PEG4000, 30 mM ammonium sulfate and 0.1 M TRIS, pH 9) solutions. Crystals appeared in one to three days in drops equilibrated over an 800 μL reservoir. Before data collection, crystals were washed in the cryoprotectant solution (20 % *vol*/*vol* PEG400, 30 % *wt*/*vol* PEG4000, 30 mM ammonium sulfate and 0.1 M TRIS, pH 9) and flash frozen in liquid nitrogen.

### 3.4. Data Collection and Processing, Structure Solution and Refinement 

X-ray crystallographic data were collected using synchrotron radiation at the Diamond Light Source (DLS, Didcot, United Kingdom) beamlines I03, I04-1, and I24, equipped with either Dectris Pilatus 6M-F or Pilatus3 6M detectors, and at the PetraIII (DESY, Hamburg, Germany) beamline P14 on a Dectris Pilatus 6M-F detector. Reflections were indexed and integrated using the program Mosflm [29] and scaled with SCALA [30] from the CCP4 suite [31]. Data collection and reduction statistics are displayed in Table S1. *Ef*TS crystals belonged to the primitive monoclinic space group P2_1_, with two enzyme dimers in the cell asymmetric unit (ASU). hTS crystals are monoclinic, space groups C2 or I2, with three enzyme subunits in the ASU (one and half hTS dimer). In this case, one hTS dimer sits on a space group special position of multiplicity 2 (two-fold axis).

The structures of the complexes were solved by molecular replacement using the software Molrep [32] from the CCP4 suite. One monomer of hTS in the active conformation (PDB id 1HWY [3]) and a functional *Ef*TS dimer (PDB id 3UWL [5]) were used as searching models, excluding water molecules and non-protein atoms. All structures were refined with Refmac5 [33] from the CCP4 suite using TLS parametrization [34] in the last cycles of the refinement protocol. The optimal partitioning of the polypeptide chains was calculated though the *TLS Motion Determination* web server [35], resulting in twenty continuous segments. The molecular graphic software Coot [36,37] was used for the manual rebuilding and modelling of missing atoms. Water molecules were added through the ARP/wARP suite [38] and checked with Coot. In all models, the occupancies of the exogenous ligands were adjusted to values resulting in atomic displacement parameters close to those of neighboring protein atoms in fully occupied sites. The stereochemical quality of the final models was checked using Coot and Procheck [39]. The overall quality of each structure has been validated though the Protein Data Bank submission procedure. Refinement statistics are reported in Table S2. Figures were generated using the molecular-graphic software CCP4mg [40]. 

### 3.5. PDB Deposition

Atomic coordinates and structure factors were deposited in the Protein Data Bank under the accession codes 6QYA (*Ef*TS–dUMP), 6QXS (*Ef*TS–FdUMP), 6QXH (hTS–dUMP), and 6QXG (hTS–FdUMP).

## 4. Conclusions

*Enterococci* are Gram-positive bacteria intrinsically resistant to various classes of antimicrobials [41,42]. In healthy people, *E. faecalis*, together with *E. faecium*, are only responsible for occasional urinary tract infections; however, they are deeply involved in healthcare-associated opportunistic infections. In recent years, vancomycin-resistant *E. faecalis* and *E. faecium* have become a serious health issue, highlighting the need to identify new antibiotics effective towards their infections. Bacterial TS enzymes represent a validated target for the development of antimicrobial drugs. In the past years, various classes of inhibitors have been explored for bacterial TSs, some of them specifically targeting *Enterococcus faecalis* TS, such as phtalimide-derivatives [43,44]. The most active phtalimide inhibitors of *Ef*TS showed selectivity towards the human counterpart enzyme [44]. Nevertheless, much larger efforts are required to achieve effective treatments. *Ef*TS is characterized by a quite peculiar behaviour, being the only bacterial TS known so far showing large asymmetry within each enzyme dimer. This has been associated with the alternate transitions between the open and closed states of its subunits. In this work, we provide new evidence to support the half-site reactivity model proposed for this enzyme. Indeed, asymmetrical ligand binding is observed in *Ef*TS complexes with both the substrate dUMP and its analogue FdUMP. Furthermore, these complexes provide new important insight into the mechanism concerning the transition between the open and closed states, providing evidence that it occurs in *Ef*TS as a bi-phasic process. The binding of the dUMP substrate triggers the closure of the loop 18–26, whereas the rearrangement of the SD, required to complete the transition to the closed state, needs the population of the cofactor site. This is particularly evident in the complex with FdUMP, in which the settlement of the substrate pocket is accompanied by the binding of 5-FTHF in the cofactor site. However, in *Ef*TS-dUMP and *Ef*TS-FdUMP complexes, the concomitant population of the substrate site in both dimer subunits was not observed. The reason behind this unique behaviour of *Ef*TS remains an open question.

The comparison with the analogous complexes obtained for the human enzyme highlights meaningful differences in ligand binding. In *Ef*TS, Trp84 displays a major contribution to cofactor binding, inducing a more compact assembly of the molecules in the catalytic cavity. On the other hand, hTS Asn112 matches the position of *Ef*TS Trp84, generating a rearrangement of both the cofactor and the substrate sites inside the cavity. 

The structure of *Ef*TS in complex with a representative phtalimide inhibitor (PDB id 4O7U [44]) showed close van der Waals contact between the six-membered aromatic ring of phtalimide and Trp84, explaining the selectivity profile of these molecules. Trp84 of *Ef*TS is also conserved in TSs of other human pathogens, such as *M. tuberculosis* and *S. aureus*, providing a clue for the design of new selective bacterial TSs inhibitors.

## Supplementary Materials

Figure S1: structural comparison of the 5-FTHF binding mode; Figure S2: Sequence alignment among TSs from different sources; Table S1: Data collection and processing statisctics; Table S2. Structure solution and refinement statistics.
