# Supplementary material for: Structural Comparison of Enterococcus faecalis and Human Thymidylate Synthase Complexes with the Substrate dUMP and Its Analogue FdUMP Provides Hints about Enzyme Conformational Variabilities
Source: Molecules. 2019 Mar 31;24(7):1257. doi: 10.3390/molecules24071257 (PMC6479881; doi:10.3390/molecules24071257)
Supplement: Supplementary file 1 [file molecules-24-01257-s001.pdf]

Structural comparison of *Enterococcus faecalis* and human thymidylate synthase complexes with the substrate dUMP and its analogue FdUMP provides hints about enzyme conformational variabilities

Cecilia Pozzi[1], Stefania Ferrari[2], Rosaria Luciani[2], Giusy Tassone[1], Maria Paola Costi[2], and Stefano Mangani[1,*]

[1] Department of Biotechnology, Chemistry and Pharmacy – Department of Excellence 2018-2020, University of Siena, via Aldo Moro 2, Siena, 53100, Italy; pozzi4@unisi.it (C.P.); giusy.tassone@unisi.it (G.T.)

[2] Department of Life Sciences, University of Modena and Reggio Emilia, Via Campi 103, 41125 Modena, Italy; sferrari591@gmail.com (S.F.); rosaria.luciani@gmail.com (R.L.); mariapaola.costi@unimore.it (M.P.C.)

* Correspondence: stefano.mangani@unisi.it (S.M.); Tel.: +39-0577-234255 or +39-0577-234252 (S.M.)

Table of contents:

Figure S1. S2
Figure S2. S3
Table S1. S4
Table S2. S5

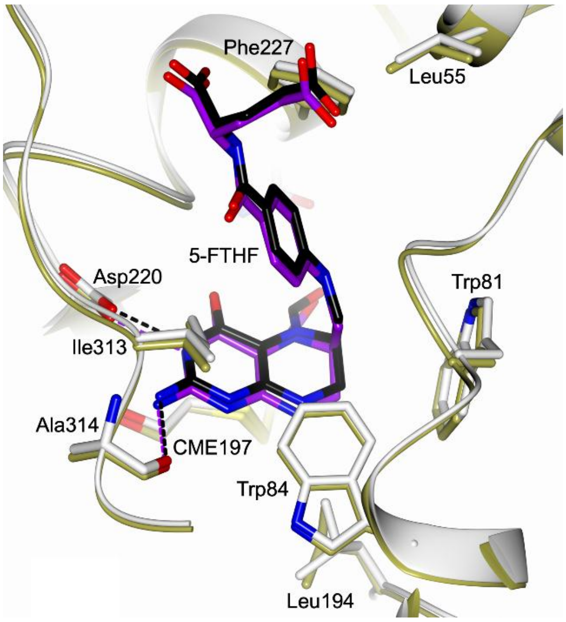

Figure S1. Active site view of the structural comparison between subunit B in the substrate-free *Ef*TS (PDB id 3UWL [5], gold cartoon and carbon atoms) and in the complex with dUMP (white cartoon and carbon atoms). In subunits B and D of both structures, the catalytic cysteine is modified as S,S-(2-hydroxyethyl)thiocysteine (CME197) and the cofactor-like molecule 5-formyl-6-tetrahydrofolate (5-FTHF, in sticks, carbon atoms are colored black and purple in the native *Ef*TS and in the complex with dUMP, respectively) occupies the active site displaying an highly conserved binding mode. H-bonds are displayed as dashed lines (colored black and purple in the native *Ef*TS and in the complex with dUMP, respectively). Oxygen atoms are colored red, nitrogen blue, sulfur yellow, and phosphorous magenta.

```
                                                                    iiiiiiiiiiiiiiii
E.faecalis TS         1 ----------------------------MEEAYLALGKKILEEGHFKEDRTGTGTYSLFG 32
L.casei TS            1 ---------------------------MLEQPYLDLAKKVLDEGHFKPDRTHTGTYSIFG 33
S.aureus TS           1 ------------------------MLNSFDAAYHSLCEEVLEIGNTRNDRTNTGTISKFG 36
C.glutamicum TS       1 --------------------------MTVPTPYEDLLRKIAEEGSHKDDRTGTGTTSLFG 34
B.melitensis TS       1 -----------------------------MRTYLDLLQHVLDHGVDRDDRTGTGTRSVFG 31
E.coli TS             1 -----------------------------MKQYLELMQKVLDEGTQKNDRTGTGTLSIFG 31
M.Tuberculosis TS       -----------------------------MTPYEDLLRFVLETGTPKSDRTGTGTRSLFG 31
human TS              1 MPVAGSELPRRPLPPAAQERDAEPRPPHGELQYLGQIQHILRCGVRKDDRTGTGTLSVFG 60
                                                        *          *    *** *** * **

                        iiii            a     a                        aa  a
E.faecalis TS           YQMRFDLAKGFPLLTTKRVPFGLIKSELLWFLKGDTNIRYLLERNNHIWDEWAFERYVKS 92
L.casei TS              HQMRFDLSKGFPLLTTKKVPFGLIKSELLWFLHGDTNIRFLLQHRNHIWDEWAFEKWVKS 93
S.aureus TS             HQLRFDLSKGFPLLTTKKVSFKLVATELLWFIKGDTNIQYLLKYNNNIWNEWAFENYIKS 96
C.glutamicum TS         QQIRFDLNEGFPLLTTKKVHFHSVVGELLWFLQGDSNVKWLQDNNIRIWNEWADE----- 89
B.melitensis TS         YQMRFDLEEGFPVLTTKKLHLRSIIHELLWFLKGDTNIAYLKENGVTIWDEWADE----- 86
E.coli TS              HQMRFNLQDGFPLVTTKRCHLRSIIHELLWFLQGDTNIAYLHENNVTIWDEWADE----- 86
M.Tuberculosis TS       QQMRYDLSAGFPLLTTKKVHFKSVAYELLWFLRGDSNIGWLHEHGVTIWDEWAS------ 85
human TS                MQARYSLRDEFPLLTTKRVFWKGVLEELLWFIKGSTNAKELSSKGVKIWDANGSRDFLDS 120
                         * *  *   **  ***         *****  *  *   *      **

                                                                    a      i
E.faecalis TS           ADYQGPDMTDFGHRVLQDPAFAEQYKEEHQKFCDAILNDAEFAEKYGELGNIYGAQWRHW 152
L.casei TS              DEYHGPDMTDFGHRSQKDPEFAAVYHEEMAKFDDRVLHDDAFAAKYGDLGLVYGSQWRAW 153
S.aureus TS             DEYNGPDMTDFGHRALSDPEFNEQYKEQMKQFKQRILEDDTFAKQFGDLGNVYGKQWRDW 156
C.glutamicum TS         ---------------------------------------------DGELGPVYGVQWRSW 104
B.melitensis TS         ---------------------------------------------NGDLGPVYGYQWRSW 101
E.coli TS              ---------------------------------------------NGDLGPVYGKQWRAW 101
M.Tuberculosis TS       --------------------------------------------DTGELGPIYGVQWRSW 101
human TS                LGFS--------------------------------------TREEGDLGPVYGFQWRHF 142
                                                                      * **  ** ***

                        ii        i      i  ii  i     iiiiiiiiiiiiiiiiiiiiiiiii  i i
E.faecalis TS           ET--------KDGSFIDQLANVIEMIKTNPDSRRLIVSAWNPEDVPSMALPPCHTMFQFY 204
L.casei TS              HT--------SKGDTIDQLGDVIEQIKTHPYSRRLIVSAWNPEDVPTMALPPCHTLYQFY 205
S.aureus TS             VD--------KDGNHFDQLKTVIEQIKHNPDSRRHIVSAWNPTEIDTMALPPCHTMFQFY 208
C.glutamicum TS         PT--------PDGRHIDQISGALETLRNNPDSRRNIVSAWNVSELENMALPPCHLLFQLY 156
B.melitensis TS         PA--------PDGRHIDQIANLLKMLHTNPQSRRLIVSAWNPALVDEMALPPCHCLFQFY 153
E.coli TS              PT--------PDGRHIDQITTVLNQLKNDPDSRRIIVSAWNVGELDKMALAPCHAFFQFY 153
M.Tuberculosis TS       PAP--------SGEHIDQISAALDLLRTDPDSRRIIVSAWNVGEIERMALPPCHAFFQFY 153
human TS                GAEYRDMESDYSGQGVDQLQRVIDTIKTNPDDRRIIMCAWNPRDLPLMALPPCHALCQFY 202
                                    *   **           *  ** *  ***      *** ***   * *

                        iii   iiiiiiiii                             i i iiii i
E.faecalis TS           VNEGKLSCQLYQRSADVFLGVPFNIASYALLTHLIAHETGLEVGEFVHTLGDAHLYQNHV 264
L.casei TS              VNDGKLSLQLYQRSADIFLGVPFNIASYALLTHLVAHECGLEVGEFIHTFGDAHLYVNHL 265
S.aureus TS             VQDGKLSCQLYQRSADIFLGVPFNIASYALLTHLIAKECGLEVGEFVHTFGDAHIYSNHI 268
C.glutamicum TS         VADGKLSCQLYQRSADMFLGVPFNIASYALLTHMFAQQAGLEVGEFIWTGGDCHIYDNHK 216
B.melitensis TS         VANGRLSCQLYQRSADIFLGVPFNIASYALLTMMIAQVTGLKPGEFIHTLGDAHIYSNHF 213
E.coli TS              VADGKLSCQLYQRSCDVFLGLPFNIASYALLVHMMAQQCDLEVGDFVWTGGDTHLYSNHM 213
M.Tuberculosis TS       VADGRLSCQLYQRSADLFLGVPFNIASYALLTHMMAAQAGLSVGEFIWTGGDCHIYDNHV 213
human TS                VVNSELSCQLYQRSGDMGLGVPFNIASYALLTYMIAHITGLKPGDFIHTLGDAHIYLNHI 262
                        *    ** ****** *  ** **********    *    *  * *  * ** * * **

                                                            a   aa
E.faecalis TS           EQMQEQLSREVRSFPTLVLNPDKASVFDFDMEDIKVEGYDPHPTIKAPIAV 315
L.casei TS              DQIKEQLSRTPRPAPTLQLNPDKHDIFDFDMKDIKLLNYDPYPAIKAPVAV 316
S.aureus TS             DAIQTQLARESFNPPTLKINSDK-SIFDINYEDLEIVDYESHPAIKAPIAV 318
C.glutamicum TS         EQVAEQLSREARPYPTLELNK-AASMFEYSFDDITVSGYDPHPLIRGKVAV 264
B.melitensis TS         EQARLQLTRTPKKLPVMHINPDVKDLFAFRFEDFRLDGYEADPTIKAPIAV 266
E.coli TS              DQTHLQLSREPRPLPKLIIKRKPESIFDYRFEDFEIEGYDPHPGIKAPVAI 264
M.Tuberculosis TS       EQVRLQLSREPRPYPKLLL-ADRDSIFEYTYEDIVVKNYDPHPAIKAPVAV 263
human TS                EPLKIQLQREPRPFPKLRILRKVEKIDDFKAEDFQIEGYNPHPTIKMEMAV 313
                             ** *     *                 *     *   * *    *
```

Figure S2. Sequence alignment among TS from different sources. Conserved residues are indicated by asterisks. The catalytic cysteine and the small domain of each TS enzyme are highlighted in cyan and orange, respectively. *Ef*TS residues belonging to the interface area and to the active site are indicated by "*i*" and "*a*", respectively.

Table S1. Data collection and processing (Values for the outer shell are given in parentheses).

	*Ef*TS – dUMP	*Ef*TS – FdUMP	hTS – dUMP	hTS – FdUMP
**PDB code**	**6QYA**	**6QXS**	**6QXH**	**6QXG**
Diffraction source	I04-1 (DLS)	I24 (DLS)	P14 (PetraIII)	I03 (DLS)
Wavelength (Å)	0.92819	0.9861	0.97625	0.97625
Temperature (K)	100	100	100	100
Detector	Dectris Pilatus 6M	Dectris Pilatus 6M	Dectris Pilatus 6M	Dectris Pilatus 6M
Crystal-detector distance (mm)	293.4	437.0	381.9	363.5
Rotation range per image (°)	0.25	0.25	0.20	0.25
Total rotation range (°)	200	160	360	300
Exposure time per image (s)	0.40	0.08	0.10	0.30
Space group	$P2_1$	$P2_1$	C2	I2
a, b, c (Å)	73.16; 95.10; 97.41	72.04; 94.84; 97.00	182.11; 61.59; 101.10	103.22; 61.89; 169.36
β (°)	93.63	94.01	115.38	98.96
Mosaicity (°)	0.51	0.75	0.36	0.55
Resolution range (Å)	73.01-1.76 (1.86-1.76)	96.76-2.88 (3.04-2.88)	34.19-2.04 (2.15-2.04)	33.37-2.08 (2.19-2.08)
Total No. of reflections	389827 (58951)	61694 (8929)	393536 (57948)	281895 (40808)
No. of unique reflections	125005 (18480)	28081 (4058)	62630 (9114)	63324 (9136)
Completeness (%)	95.1 (96.5)	95.1 (94.7)	97.0 (97.2)	99.4 (98.8)
Redundancy	3.1 (3.2)	2.2 (2.2)	6.3 (6.4)	4.5 (4.5)
$\langle I/\sigma(I)\rangle$	6.9 (2.9)	4.2 (2.0)	12.8 (3.3)	7.5 (2.0)
R_{meas}	0.131 (0.425)	0.198 (0.466)	0.072 (0.528)	0.103 (0.617)
Overall B factor from Wilson plot ($Å^2$)	10.40	30.71	43.18	37.05

Table S2. Structure solution and refinement (Values for the outer shell are given in parentheses).

	*Ef*TS – dUMP	*Ef*TS – FdUMP	hTS – dUMP	hTS – FdUMP
**PDB code**	**6QYA**	**6QXS**	**6QXH**	**6QXG**
Resolution range (Å)	68.07-1.76 (1.81-1.76)	96.76-2.88 (2.96-2.88)	34.22-2.04 (2.09-2.04)	32.36-2.08 (2.13-2.08)
Completeness (%)	94.88 (96.44)	94.42 (95.20)	96.69 (96.57)	97.86 (98.68)
No. of reflections, working set	118686 (8891)	26635 (1942)	58183 (4281)	59309 (4378)
No. of reflections, test set	6295 (471)	1350 (119)	4434 (310)	3181 (243)
Final R_{cryst}	0.184 (0.230)	0.200 (0.291)	0.187 (0.357)	0.181 (0.277)
Final R_{free}	0.220 (0.258)	0.252 (0.296)	0.237 (0.339)	0.225 (0.303)
Estimated error on coordinated based on R value (Å)	0.13	0.45	0.19	0.19
No. of non-H atoms				
Protein	9894	9670	6804	6786
Ion	16	10	6	5
Ligand	140	110	67	63
Water	1523	327	205	413
Total	11573	10117	7082	7267
R.m.s. deviations				
Bonds (Å)	0.022	0.007	0.010	0.012
Angles (°)	2.271	1.613	1.940	2.010
Average B factors (Å^2)	24.67	38.77	69.4	82.93
Ramachandran plot				
Most favoured (%)	97.9	95.2	96.9	96.5
Allowed (%)	2.1	4.8	3.1	3.5
